# Setting clinically relevant thresholds for the notification of canine disease outbreaks to veterinary practitioners: an exploratory qualitative interview study

**DOI:** 10.3389/fvets.2024.1259021

**Published:** 2024-02-28

**Authors:** Carmen Tamayo Cuartero, Eszter Szilassy, Alan D. Radford, J. Richard Newton, Fernando Sánchez-Vizcaíno

**Affiliations:** ^1^Bristol Veterinary School, Faculty of Health Sciences, University of Bristol, Bristol, United Kingdom; ^2^Bristol Medical School, Faculty of Health Sciences, University of Bristol, Bristol, United Kingdom; ^3^Institute of Infection, Veterinary and Ecological Sciences, University of Liverpool, Liverpool, United Kingdom; ^4^Department of Veterinary Medicine, Cambridge Veterinary School, University of Cambridge, Cambridge, United Kingdom

**Keywords:** disease surveillance, canine diseases, qualitative research, outbreak detection, outbreak reporting

## Abstract

**Introduction:**

The Small Animal Veterinary Surveillance Network (SAVSNET) has developed mathematical models to analyse the veterinary practice and diagnostic laboratory data to detect genuine outbreaks of canine disease in the United Kingdom. There are, however, no validated methods available to establish the clinical relevance of these genuine statistical outbreaks before their formal investigation is conducted. This study aimed to gain an actionable understanding of a veterinary practitioner’s preferences regarding which outbreak scenarios have a substantial impact on veterinary practice for six priority canine diseases in the United Kingdom.

**Methodology:**

An intensity sampling approach was followed to recruit veterinary practitioners according to their years of experience and the size of their practice. In-depth semi-structured and structured interviews were conducted to describe an outbreak notification and outbreak response thresholds for six canine endemic diseases, exotic diseases, and syndromes. These thresholds reflected participants’ preferred balance between the levels of excess case incidence and predictive certainty of the detection system. Interviews were transcribed, and a thematic analysis was performed using NVivo 12.

**Results:**

Seven interviews were completed. The findings indicate higher preferred levels of predictive certainty for endemic diseases than for exotic diseases, ranging from 95 to 99% and 80 to 90%, respectively. The levels of excess case incidence were considered clinically relevant at values representing an increase of two to four times in the normal case incidence expectancy for endemic agents, such as parvovirus, and where they indicated a single case in the practice’s catchment area for exotic diseases such as leishmaniosis and babesiosis.

**Conclusion:**

This study’s innovative methodology uses veterinary practitioners’ opinions to inform the selection of a notification threshold value in real-world applications of stochastic canine outbreak detection models. The clinically relevant thresholds derived from participants’ needs will be used by SAVSNET to inform its outbreak detection system and to improve its response to canine disease outbreaks in the United Kingdom.

## 1 Introduction

One of the main factors that determine the effectiveness of an epidemic response is the timely detection and notification to farm owners whose animals are potentially affected (1). In the United Kingdom, surveillance systems in farm animals and public health are run centrally by government departments and agencies to identify increasing disease trends and detect disease outbreaks in their early stage, facilitating the prevention and control of health threats nationally and regionally (2, 3). The relevant information derived from these surveillance activities is shared with the public via weekly reports (4) and online dashboards (5). However, these surveillance protocols do not currently exist in small companion animals, for which there is no standardized system of disease reporting or routine collection of surveillance data at a national level. This leaves canine populations in the United Kingdom vulnerable to epidemic threats.

To begin to bridge this gap, the Small Animal Veterinary Surveillance Network (SAVSNET)-Agile initiative (6) is developing a nationwide system for the timely detection and response to canine disease outbreaks in the United Kingdom. However, before such a surveillance and control system can be set up and implemented, it is necessary to determine which notification thresholds of increased levels in case incidence relative to a previously identified baseline of expected cases would warrant alerting relevant stakeholders of potential outbreak threats.

There are several methods that have been described to determine statistical outbreak notification thresholds. These methods vary depending on the disease type and the quality of the data that is available for surveillance purposes. For diseases that are endemic to the country, systems rely on historical data to establish a baseline level of disease and then use different mathematical methods to determine notification thresholds based on increases in case incidence, relative to the previously identified baseline (7, 8). Other commonly used methods to establish outbreak notification thresholds are multi-chart schemes, which combine the results of an individual time series that enable the rapid detection of subtle changes in the disease (8) or the methods that involve setting the number of standard deviations above the baseline of expected cases (9). For exotic and rare diseases, due to a lack of baseline data to draw patterns from, notification thresholds are defined *ad hoc*, and it is often common to accept a single case as a threat that warrants generating an alert (10).

While these statistical methods have proven to be powerful for detecting disease anomalies, they often signal outbreaks that are not clinically relevant for veterinarians in practice. Therefore, outbreak notification systems that rely on such statistical signals might overload practitioners with information that is not actionable. In the long term, this overloading could lead to a lack of confidence and engagement with the surveillance and outbreak notification system. To address these limitations, this study aimed to explore what threshold values based on veterinary practitioners’ opinions correspond to outbreaks that should be notified when detected by the statistical methods because of their significant impact on veterinary practice for six priority canine diseases in the United Kingdom (11). In addition, we gained an understanding of the reasons that drive veterinary practitioners to select such threshold values and of how their in-practice behavior can be impacted by clinically relevant outbreaks. To achieve these aims, an innovative methodology was developed based on the combination of semi-structured and structured interviews with companion animal veterinarians.

## 2 Materials and methods

Ethical approval for this study was granted by the ethics committee of the University of Bristol Faculty of Health Sciences (FREC, reference code: 98843).

### 2.1 Study population

The population of interest for this study was small animal veterinary clinicians working in the United Kingdom at the time of its conduction. Study participants were selected from this population following an intensity sampling approach, which is a type of purposive sampling to select information-rich cases located at the end of a population’s distribution (12). To select information-rich cases, relevant population characteristics, or descriptors, were defined. These descriptors were believed to influence participants’ perspectives and behavior regarding canine epidemics and, therefore, their responses during the interviews. The following descriptors and levels of interest were used in the sampling process to categorize recruited participants:

(a) Years of experience in small animal practice: It was assumed that more senior veterinarians are more likely to have experienced canine outbreaks throughout their career and have spent more time in practice overall, which could influence their opinions and decision-making. The cutoff points were established to differentiate newly graduated veterinarians from those with many years of in-practice experience.
  (a) Recent graduates: Those with less than 5 years of experience.
  (b) Senior veterinarians: Those with over 10 years of experience.

(b) Practice size: Since smaller practices have fewer employed veterinarians and see a lower number of cases, compared to bigger veterinary centers, it was expected that an outbreak would affect them differently and could potentially overwhelm their ability to cope with the increase in case incidence. To accurately reflect the difference between small and big veterinary practices, a summary of the existing veterinary practices in the United Kingdom was requested from the Royal College of Veterinary Surgeons (RCVS). This database included the total number of registered practices in the United Kingdom and a breakdown of the number of employed veterinarians per practice. The practice directory was analyzed to understand what the average size of the practice is and inform the categorization. A total of 4,252 individual veterinary sites were listed on the database. Over half of these sites had four or fewer registered veterinary surgeons (2,917 or 68%). A total of 23% (984) of the sites had between five and nine employed veterinarians, and only a small number (348 or 8%) had 10 or more registered veterinary surgeons.
  (a) Small veterinary practice: Those with fewer than four employed veterinarians.
  (b) Large veterinary practices: Those with more than 10 employed veterinarians.

### 2.2 Participant recruitment

Participant recruitment was conducted from July 2021 to April 2022. Potential study participants were contacted through different means. Veterinary clinicians who were part of a pre-established network of collaborators for SAVSNET-Agile were emailed directly by the corresponding author (CTC). Furthermore, veterinary practices that contributed data to SAVSNET at the time of the conduction of the study were contacted via email and their practice management software (PMS); these practices contain a SAVSNET plugin window that can be used by the latter to relay messages to attending veterinarians (13). A participant recruitment advertisement was posted on the SAVSNET website (14) and shared on social media, including on Twitter and Facebook. Finally, an interview to advertise the study was conducted by the corresponding author (CTC) with the United Kingdom veterinary magazine, Vet Times (15).

### 2.3 Interviews with companion animal veterinarians

Recruited veterinarians took part in an interview session, which was conducted online via Microsoft Teams (16) or Zoom (17). Interviews were conducted between August 2021 and April 2022. The overall aim of the interviews was to explore clinically relevant outbreak scenarios for the notification of two canine endemic diseases (leptospirosis and parvovirus), two canine exotic diseases (leishmaniosis and babesiosis), and two canine syndromes (respiratory and gastrointestinal diseases). The interviews consisted of two components, with different aims.

#### 2.3.1 Semi-structured interview

The first part of the interview followed a semi-structured (18), in-depth format and aimed to gain an understanding of the reasons that drive veterinary practitioners to define what constitutes a clinically relevant outbreak and to understand how their in-practice behavior can be impacted by such outbreaks. To facilitate the discussion, the interviewer first provided an overview of the epidemiological characteristics of the disease under consideration. The topic guide developed for the semi-structured interview can be found in Supplementary material S1. When participants did not know or had misconceptions about the characteristics of a particular disease, these doubts or misconceptions were clarified by the interviewer at the end of the interview session.

#### 2.3.2 Structured interview

Once participants had reflected upon the subject matter, the interview changed to a structured format to understand which outbreak scenarios would be selected by participants to receive timely alerts due to their potential impact on their practice. Outbreak scenarios were described using two parameters, which represented the characteristics of an outbreak notification:

- Excess case incidence: An increased incidence above the expected baseline of cases in your practice’s catchment area would be of practical significance to (a) warrant a notification about a potential outbreak and (b) drive you to change your behavior in practice in response to an outbreak. Thus, when selected levels of excess case incidence were different for (a) and (b), the selected value for the former was used to define a notification threshold, and the value for the latter was used to define an outbreak response threshold for canine diseases.
- Predictive certainty: The level of confidence of the alerts generated by the statistical outbreak detection models, defined by their credible interval, normally takes values that range from 90 to 99% (19).

The questions included in the structured interview (Supplementary material S2) aimed to introduce the concepts of excess case incidence and predictive certainty to study participants and use them to describe disease-specific outbreak scenarios in a way that resonated with participants and their experience in practice.

### 2.4 Data analysis

**Figure 1:**
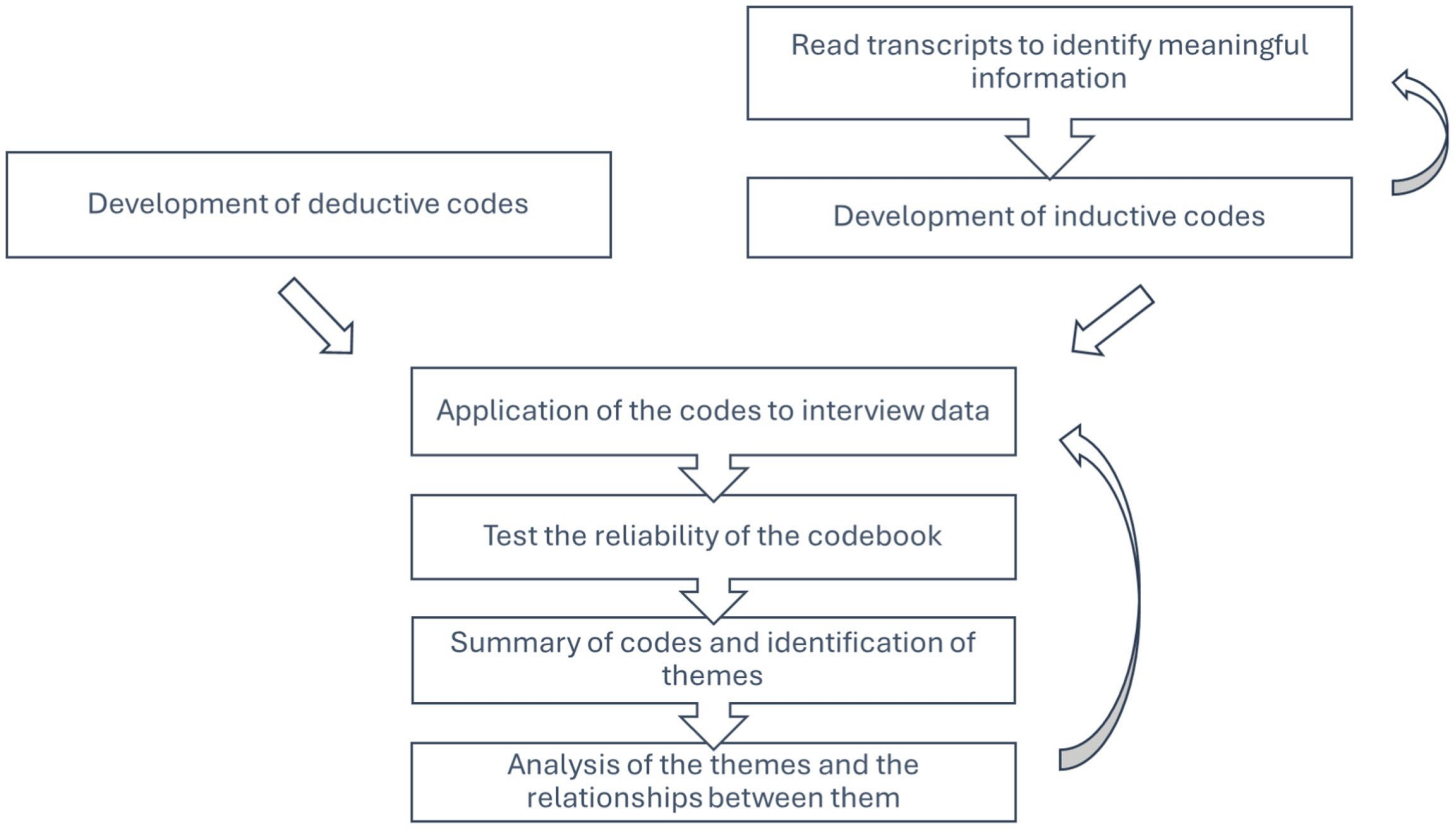
A summary of the data analysis process for veterinary clinicians’ interview transcripts.

Interview data were audio-recorded and transcribed verbatim. All the analyses were conducted on NVivo (version 12) qualitative data analysis software (20). A coding framework was iteratively developed by the corresponding author (CTC) based on the expected and emergent themes using deductive and inductive approaches, respectively. To enhance the consistency and reliability of the analysis, two authors (CTC and FSV) independently coded the transcript data from one of the interviews. Codes generated deductively and inductively from interview transcripts were grouped together into themes by following a hybrid approach to thematic analysis (21, 22) (Figure 1). To ensure reliability and transparency, themes were continuously compared to the interview transcripts to ensure they were true to the original data (23).

## 3 Results

### 3.1 Characteristics of participants

Seven veterinary clinicians participated in this study. Out of these participants, four were part of SAVSNET’s previously established network of collaborators, two had seen the recruitment advertisement on SAVSNET’s PMS plugin window, and one reported having seen the study in an online veterinary magazine. Table 1 presents a summary of the characteristics of the recruited participants, according to the study’s population descriptors. Five out of the seven participants had more than 10 years of experience in practice, and the remaining two participants had worked in small animal practices for less than 5 years. Four of the participants were employed by large veterinary centers, with more than 10 veterinary surgeons, while the other three worked in small clinics, with fewer than five veterinary surgeons. In fact, in the latter case, two participants (numbers 2 and 6) worked in centers where a single veterinary surgeon was on duty at any given time.

**Table 1:** The breakdown of participating veterinary clinicians, with a breakdown of their characteristics according to the population descriptors of the study.

Participants	Practice size (In no. of employees)	Experience (In years)
1	4	32
2	3	18
3	80	14
4	14	25
5	23	16
6	2	1.5
7	11	4

### 3.2 Findings from interviews with veterinary clinicians

The codebook used to analyze interview transcript data can be found in Supplementary Table S3. Interviews had a mean duration of 1 h 10 min, the longest one being 1 h 34 min and the shortest being 50 min.

The results of this study are presented as follows: first, an overview of the excess case incidence and predictive certainty parameters was summarized, and second, for each of the diseases under study, themes that resulted from grouping inductive and deductively generated codes as well as the values chosen by participants for the outbreak notification and outbreak response thresholds were shown.

#### 3.2.1 Excess case incidence

When discussing the levels of excess incidence to define notification and outbreak response thresholds, some participants preferred to discuss this parameter by providing a single value of disease case incidence that would make them want to either be notified about a potential outbreak in their area or—in addition to this notification—also change their in-practice behavior. In other cases, especially if they had never personally dealt with the disease in question, participants felt more comfortable discussing the excess incidence as a range of the values of case incidence. Participants also had different preferences for the time unit used to discuss the excess incidence, e.g., some participants referred to an increase in case incidence within a week or a month, while others simply provided an absolute number of disease cases. Furthermore, some participants discussed the excess incidence as an increase in the number of cases relative to the expected baseline, e.g., two or three times higher than expected, while others preferred to provide an absolute number of cases that would warrant a notification or that would trigger a behavior change in their practice.

#### 3.2.2 Predictive certainty

The predictive certainty parameter was interpreted by participants in two distinct, opposite ways. On the one hand, some participants expressed that they would rather set the predictive certainty value at the lowest possible level when dealing with diseases that they considered as posing a high epidemic risk. They argued that they would rather be notified as soon as possible about severe potential threats to increase their practice’s preparedness, despite the higher probability of receiving a false alert. Conversely, other participants preferred to set the predictive certainty value to the highest level when faced with the same situation. Their rationale was that, given the high severity of the disease threat, the participants would only require a notification if the risk of receiving a false alert is minimized in order to avoid either wasting time and resources in preparing for a non-existent epidemic or unnecessarily warning the practice’s clients. This scenario was reflected, for example, in the case of canine leptospirosis, which was perceived as a very severe, life-threatening disease, for which some participants chose relatively low predictive certainty values (90%), while others set this parameter value at 99%.

#### 3.2.3 Canine leptospirosis

Overall, participants perceived canine leptospirosis as the pathogen that posed the highest epidemic risk to their practices, mainly due to the uncertainties surrounding the disease’s diagnosis, treatment, and prevention.

##### 3.2.3.1 Diagnostic challenges

A recurring theme that emerged from the interviews was the challenges and uncertainties surrounding the diagnosis of canine leptospirosis. Although participants were aware of the different diagnostic tools that can be used to diagnose the disease, they were unsure of which tests to use to ensure the reliability of the results, depending on the stage of the infection.

*“You’re gonna end up with more questions than answers from me on this, because I still think there’s an awful lot to be answered diagnostically, um, on lepto.”*

*— Participant 4: 25 years of experience, practice of 14 veterinarians.*

Another source of diagnostic uncertainty was the variety of clinical presentations of leptospirosis. Those participants who had been involved in an outbreak in the past recalled how the cases of confirmed leptospirosis they had did not show the signs commonly associated with this disease. Furthermore, the rapid progression of the disease means that it is sometimes difficult to perform diagnostic tests or take samples to confirm the diagnosis.

*“[leptospirosis] is very acute, the animal died in a couple of days…. So yeah, we did not even have time to perform more tests*.”

*— Participant 7: 4 years of experience, practice of 11 veterinarians.*

Participants also discussed the difficulty posed by carriers that can spread the disease despite not showing any clinical signs. Due to these diagnostic barriers, only two participants had ever reached a definitive diagnosis of canine leptospirosis throughout their careers, while other participants had only seen highly suspicious, yet unconfirmed, potential cases.

*“[…] our diagnosis was empiric, it was a diagnosis just based on clinical signs, we did not go any further diagnostic-wise […] and it was a dog living in a farm, so all of this made us suspicious.”*

*— Participant 7: 4 years of experience, practice of 11 veterinarians.*

##### 3.2.3.2 Vaccination

Study participants often discussed the vaccination practices for canine leptospirosis and highlighted key issues regarding leptospirosis vaccines. They perceived these issues as an important obstacle for the adequate prevention of this disease. For instance, participants were unsure of the length of the immunity provided by leptospirosis vaccines, the frequency of vaccinations that they should recommend to dog owners, and how to convey the importance of vaccination to their clients.

*“I would love to know how long lepto immunity lasts in the system, the same way you can do a titre test for dhp […] but I’d like to have a way of knowing more accurately how long the immunity lasts in the dog’s body… any kind of approach to know how protected the dog is against lepto.”*

*— Participant 6: 1.5 years of experience, practice of 2 veterinarians.*

Furthermore, vaccine hesitancy was reported to be the highest among veterinary professionals and dog owners in the case of canine leptospirosis. The vaccine hesitancy was mainly related to the controversies associated with the relatively newly introduced L4 vaccine (a quadrivalent canine leptospirosis vaccine named Novibac L4® by Merck & Co., Inc.).

*“Leptospirosis is one that is part of our core vaccines, and we use nobivac so it’s the infamous leptospirosis 4, which obviously carries all the interesting discussions that go with it, probably similar to covid and 5G.”*

*— Participant 7: 4 years of experience, practice of 11 veterinarians.*

##### 3.2.3.3 Zoonotic risk

Study participants were either unaware of the zoonotic potential of leptospirosis or did not believe this pathogen to pose a relevant risk to humans. Only one participant recounted observing a potential dog-to-owner transmission of leptospirosis during their career:

“*One dog, we had referred a Jack Russell a number of years ago, the owner died of leptospirosis. Um, the dog had leptospirosis, so we have seen that once.*”

*— Participant 3: 14 years of experience, practice of 80 veterinarians.*

##### 3.2.3.4 Clinically relevant threshold

**Figure 2:**
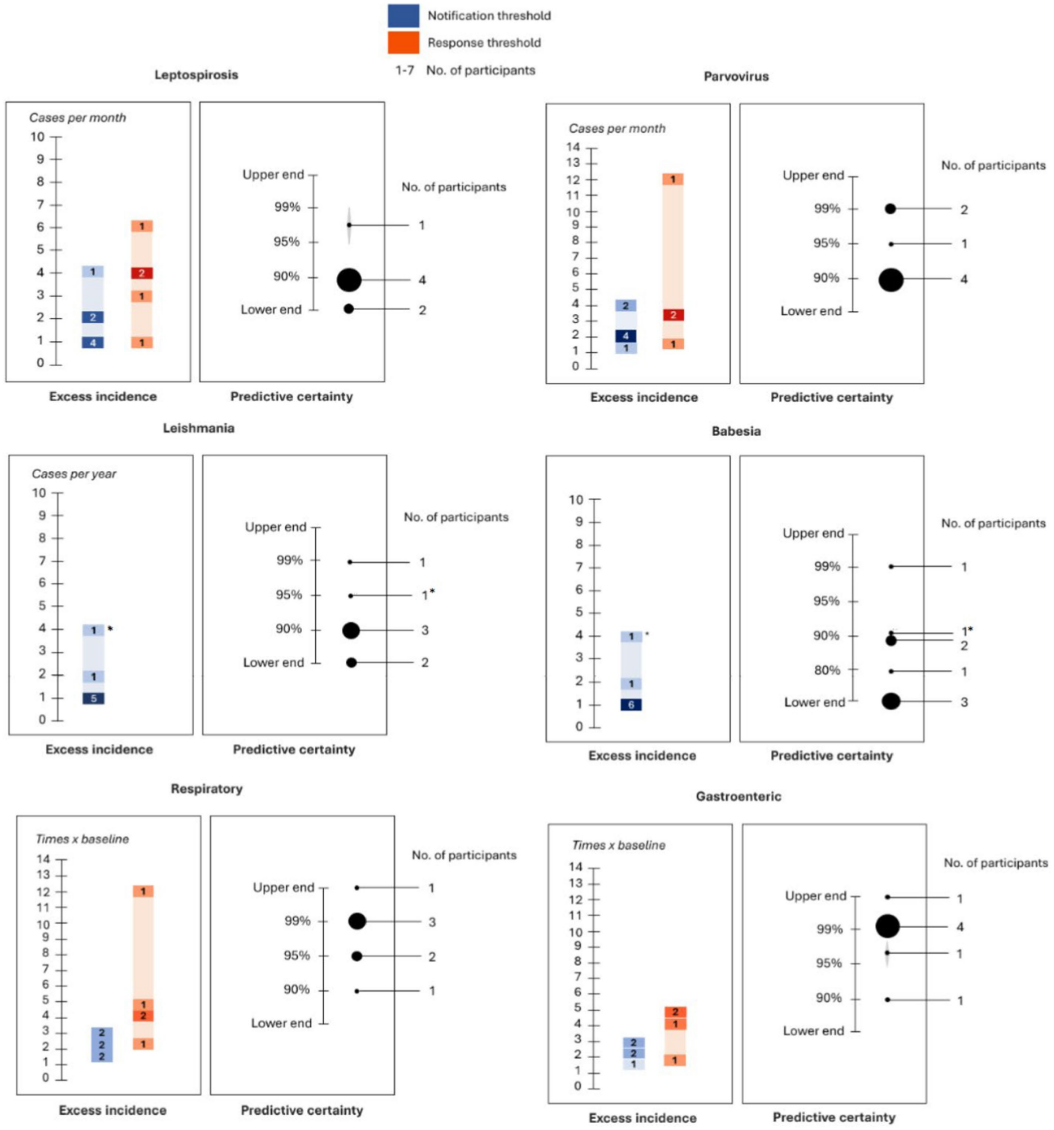
A summary of clinically relevant thresholds for the six canine diseases included in this study. The left graph included for each disease depicts the excess case incidence thresholds. The blue and orange bars represent the excess incidence values corresponding to the notification and outbreak response thresholds, respectively. If the same participant provided more than one value of case incidence to define either the notification threshold or the outbreak response threshold, then the lowest value was used to depict the former, while the highest value was used for the latter. These bars are rendered using a gradient of colors, which serve as an indicator of the number of participants who provided a particular value, with darker colors indicating a higher number of participants. For each disease, the right graph depicts the overall predictive certainty threshold as the range of values provided by participants. The size of the dots corresponds to the number of participants who provided that particular value, with larger dots indicating a higher number of participants. For exotic diseases, the asterisk denotes responses that were specific to non-autochthonous cases of the disease, and the other responses refer to autochthonous cases.

Figure 2 shows an overview of the clinically relevant notification threshold values for canine leptospirosis, including the notification and outbreak response thresholds. When discussing the clinically relevant threshold for canine leptospirosis, most participants would like to be notified as soon as a single case was detected in their area (Table 2). Moreover, some participants enquired about the surveillance system’s capacity to flag highly suspicious cases, even without an official diagnosis and account for “leptospirosis-like illness,” given the existing diagnostic difficulties. For this reason, all but one of the participants preferred to set the predictive certainty of alerts to low levels (Table 3). The only participant who did not agree was one of the two veterinarians who had been involved in a past leptospirosis outbreak, who would rather be notified only if the certainty level was very high, given the high levels of distress among employees and clients and the investment in resources for preparedness:

**Table 2:** A summary of participants’ preferred levels for the notification and outbreak response thresholds and predictive certainty values for canine leptospirosis.

Canine leptospirosis				
Participants	Baseline of cases in their practice	Notification threshold	Outbreak response threshold	Predictive certainty values
	0 / month Seen 3 times throughout the career	1 case/month	N/A	90%
2	0 cases/ month Seen 2 cases	1 case/month	3 cases/month	90%
3	2–3 cases/year Involved in outbreak	2–3 cases/month	4 cases/month	90%
4	1–3 cases/year Involved in outbreak	x2 baseline/ month	x3-4 baseline/ month	90%
5	0 cases/month Involved in outbreak	1 case/week	2–3 cases/fortnight	95–99%
6	0 cases/month Seen 1 case (never confirmed)	2 cases/month	4 cases/month	The lowest end of the possible range
7	0 cases/month Seen 2 cases (never confirmed)	>0 case/month	N/A	The lowest end of the possible range

The table includes participant’s reported baseline of observed cases in their practices.

**Table 3:** A summary of participants’ preferred levels for the notification and outbreak response thresholds and predictive certainty values for canine parvovirus.

Canine parvovirosis				
Participants	Baseline of cases in their practice	Notification threshold	Outbreak response threshold	Predictive certainty values
1	3–4 cases/year Expects certain prevalence	4 cases/month	N/A	90%
2	Expects certain prevalence throughout the year	2 cases/month	3 cases/month	90%
3	3–4 cases/year Common to hear about outbreaks	2x baseline	3–4x baseline	95%
4	Expects certain prevalence Common to hear about outbreaks	2 cases/month	12 cases/month	90%
5	Frequently seen	4 cases/month	N/A	90%
6	Expects certain prevalence Common to hear about outbreaks	2x baseline	3–4x baseline	99%
7	2–3 cases/year	2 cases/month	N/A	Closer to 99%

The table includes participants’ reported baseline of observed cases in their practices.

*“I think that a false alarm would be quite detrimental because of my experience of knowing how involved we got with this last time. I think you would want to have a relatively high level of certainty with this disease. We would have to be a bit careful that we did not create a massive scare around this and put all of this effort in, to then have clients be a bit angry and upset that we have done all of that and actually, it was just a false alarm.”*

*— Participant 4: 25 years of experience, practice of 14 veterinarians.*

#### 3.2.4 Canine parvovirus

##### 3.2.4.1 Relevance of disease

Out of the evaluated diseases, canine parvovirus is a disease that is most often seen by participants in veterinary practice. Parvovirosis is perceived as a very severe disease that appears as a peracute infection and is very intensive to treat. The participants also perceived canine parvovirus as more transmissible than canine leptospirosis among the canine population. Consequently, participants agreed about the relevance of parvovirus and did not consider it as a lesser threat for its lack of zoonotic potential:

*“Parvovirus is severe enough that I think it warrants an active response. Just because it does not affect people does not mean it’s not important, you know, there’s a significant proportion of affected dogs.”*

*— Participant 4: 25 years of experience, practice of 14 veterinarians.*

##### 3.2.4.2 Transmissibility

Participants mentioned how canine parvovirus was particularly concerning given the risk of transmission within a veterinary practice. It is notable that parvovirus was the only pathogen for which some participants reported having a pre-established protocol in their practice:

*“Our practice protocol is extremely tight. Anything that arrives at the practice that even looks like it may be parvo, a staff member will go out and a sample in the car park and the client will wait in their car with their puppy until we know it’s negative, so we know whether we are taking them and putting them straight in isolation or what we are doing with them.”*

*— Participant 5: 16 years of experience, practice of 23 veterinarians.*

##### 3.2.4.3 Risk factors

Participants discussed risk factors that they believed were associated with parvovirus. Most of them mentioned how it usually affects puppies and unvaccinated dogs, while the remaining participants also mentioned other factors that they considered relevant, such as the socioeconomic background of the owners. The factor of socioeconomic background sparked some strong opinions during the interviews, while some participants believed there to be a link between the owner’s background and the disease:

“Where I used to work, it was a rougher area, so we tended to see little outbreaks then. I think there was a particular set of clientele… what’s the right word? *long pause* sort of poorer families? They did not vaccinate and get dogs from not necessarily good areas so I think that’s why it tended to sight through a bit more.”

*— Participant 2: 18 years of experience, practice of 3 veterinarians.*

However, other participants disagreed and even had a strongly negative reaction when probed about this idea.

##### 3.2.4.4 Clinically relevant thresholds

Study participants preferred higher notification and outbreak response thresholds for canine parvovirus compared to canine leptospirosis (Table 3). The predictive certainty values chosen for parvovirus were the highest among all the specific pathogens included in this study for some participants (Table 3), given the higher prevalence of disease and the ease of diagnosis of canine parvovirus. Figure 2 shows an overview of the clinically relevant notification threshold values for canine parvovirus.

*“Parvovirosis nowadays, it’s so easy to be certain, you do a snap test, takes you 5 min to know, they are quite accurate those types of tests. So, I think, in this case, I’d prefer to know with more certainty.”*

*— Participant 7: 4 years of experience, practice of 11 veterinarians.*

#### 3.2.5 Canine leishmaniosis

##### 3.2.5.1 Knowledge about the disease

Most study participants had knowledge about the transmission routes and transmission vector of canine leishmaniosis. However, some of them were not aware of the epidemiological characteristics of the disease, and some misconceptions were also identified.

*“I’m worried because positive dogs can spread it to another dog just by skin contact […] and it’s a zoonotic disease, it can be transmitted to people from their dogs through skin lesions.”*

*— Participant 6: 1.5 years of experience, practice of 2 veterinarians.*

##### 3.2.5.2 Risk of entry in the United Kingdom

Some participants were greatly concerned about this pathogen entering the United Kingdom and spreading in the local canine population, which they believed was inevitable due to factors such as climate change and globalization. Participants also shared some strong opinions about the current dog importation practices into the country and how they exacerbate their concerns about the entry of exotic pathogens.

*“It makes me really uncomfortable, that people think it’s a wonderful idea to import dogs from Romania and from elsewhere […] there seems to be this mass push for charities and organisations to bring them in. I personally think it’s a really bad idea to be importing dogs that have or are at risk of having a disease that we do not have. What we are doing really is creating a reservoir of a zoonotic disease that we did not previously have.”*

*— Participant 5: 16 years of experience, practice of 23 veterinarians.*

Conversely, other participants argued that leishmania does not pose a risk for the canine population in the United Kingdom, since the vector is not currently present in the country.

*“How do I respond to an outbreak of canine leishmaniosis? I do not believe canine Leishmania exists as an outbreak disease.”*

*— Participant 4: 25 years of experience, practice of 14 veterinarians.*

##### 3.2.5.3 Clinically relevant threshold

Most participants had seen chronic cases of leishmaniosis in their practice, although only two of them, namely, participants 3 and 5, had ever diagnosed a case in the United Kingdom (see Table 4). The excess incidence notification threshold for leishmaniosis was over zero cases for all the participants, although some of them specified that they would only want to receive a notification if the cases were autochthonous or if the disease vector became endemic in the country (Table 4). Participants did not provide an outbreak response threshold for this exotic disease as the notification threshold would be enough for them to change their in-practice behavior. Five participants preferred to set the predictive certainty values for leishmania to the lowest possible level, whereas the remaining two participants took the opposite approach and preferred to only receive a notification if the risk of receiving a false alarm was minimized (Table 4). Figure 2 shows an overview of the clinically relevant notification threshold values for canine leishmaniosis.

**Table 4:** A summary of participants’ preferred levels for the notification and outbreak response thresholds and predictive certainty values for canine leishmaniosis.

Canine leishmaniosis				
Participants	Baseline of cases in their practice	Notification threshold	Outbreak response threshold	Predictive certainty values
1	0 seen or diagnosed	1 case/year	N/A	90%
2	Seen cases but none personally diagnosed	1 case/year	N/A	The lowest end of the possible range
3	1–2/year referred	2 cases/year (if autochthonous) 4 cases/year (if not autochthonous)	N/A	95% traveled 90% untravelled
4	Seen cases but none personally diagnosed	No notification unless the vector is present in the United Kingdom	N/A	No notification unless the vector is present
5	Seen and diagnosed cases	1 case/year	N/A	90%
6	Seen cases but none personally diagnosed	1 case/year (if autochthonous)	N/A	99%
7	Seen cases but none personally diagnosed	1 case/year (if autochthonous)	N/A	The lowest end of the possible range

The table includes participants’ reported baseline of observed cases in their practices.

#### 3.2.6 Canine babesiosis

##### 3.2.6.1 Knowledge about the disease

According to participants, canine babesiosis was even rarer than leishmaniosis as they had never seen these cases in first-opinion practice. Some participants were even surprised to hear that babesiosis could affect dogs as they had only heard about it in the context of large animals:

*“No clue about babesia in dogs, I have only seen it or studied it in horses. I’ve never even heard about it in dogs, no one has ever mentioned babesia to me.”*

*— Participant 6: 1.5 years of experience, practice of 2 veterinarians.*

Participants were doubtful about the disease’s transmission and clinical presentation, and misconceptions were identified about its zoonotic potential. When asked, participants did not believe that the knowledge of canine babesiosis among the veterinary profession in the United Kingdom is currently sufficient to adequately prevent, treat, or control the disease if an outbreak occurred.

##### 3.2.6.2 Risk of endemisation

Those participants with knowledge about canine babesiosis were greatly concerned about the possibility of endemisation of this disease in the United Kingdom, given that the tick species that can carry *Babesia* spp. are currently present in the country.

*“Babesia in untraveled dogs, I think it would be the most alarming disease. I think it’s probably only a matter of time as well, if we have already got the vector that once we introduce the pathogen it becomes established in the dog population and becomes established in those ticks.”*

*— Participant 5: 16 years of experience, practice of 23 veterinarians.*

##### 3.2.6.3 Clinically relevant threshold

Most participants considered a single case of canine babesiosis in their area enough to receive a notification and chose to set the predictive certainty value at its lowest possible level (Table 5). Participants did not provide an outbreak response threshold for this exotic disease as they considered the notification threshold enough for them to change their behavior in practice. Figure 2 shows an overview of the clinically relevant notification threshold values for canine babesiosis.

**Table 5:** A summary of participants’ preferred levels for the notification and outbreak response thresholds and predictive certainty values for canine babesiosis.

Canine babesiosis				
Participants	Baseline of cases in their practice	Notification threshold	Outbreak response threshold	Predictive certainty values
1	0 seen	1 case/year	N/A	90%
2	0 seen	1 case/year	N/A	The lowest end of the possible range
3	3 cases seen	2 cases/year (if autochthonous) 4 cases/year (if not autochthonous)	N/A	90% traveled 80% untravelled
4	Seen cases but none personally diagnosed	1 case/year	N/A	99%
5	0 seen	1 case/year	N/A	The lowest end of the possible range
6	0 seen	1 case/year	N/A	90%
7	0 seen	1 case/year	N/A	The lowest end of the possible range

The table includes participants’ reported baseline of observed cases in their practices.

#### 3.2.7 Respiratory and gastroenteric diseases

##### 3.2.7.1 Prevalence

The reported prevalence of canine syndromes was much higher than that of specific pathogens. The baseline of respiratory cases ranged from 3 to 7% of total consultations in first-opinion centers and up to 15% in a referral center (Table 6). The reported prevalence of gastroenteric disease ranged from 10 to 15% in first-opinion practice and up to 40–50% in referral centers (Table 7).

**Table 6:** A summary of participants’ preferred levels for the notification and outbreak response thresholds and predictive certainty values for canine respiratory disease.

Respiratory disease				
Participants	Baseline of cases in their practice	Notification threshold	Outbreak response threshold	Predictive certainty values
1	2 cases/day or 10–15 cases/week	2x baseline	4x baseline	99%
2	3–5% of total consultations (Total of 80 consults/week)	2x baseline (10 cases/week)	12x baseline (50 cases/week)	90%
3	10–15% of total consultations or (Total of 50 consults/week)	1.6x baseline (8/week)	2x baseline (10/week)	95%
4	Unable to provide a number, but lower than GI syndrome	+20% case increase	N/A	The upper end of the possible range
5	5–7% of total consultations 2 cases/week	3x baseline	5x baseline	95%
6	3–5% of total consultations	3x baseline	4x baseline	99%
7	3–5% of total consultations 2 cases/week	Very high increase over the baseline	N/A	Closer to 99%

**Table 7:** A summary of participants’ preferred levels for the notification and outbreak response thresholds and predictive certainty values for canine gastrointestinal disease.

Gastrointestinal				
Participants	Baseline of cases in their practice	Notification threshold	Outbreak response threshold	Predictive certainty values
1	4 cases/day or 20 cases/week	2–3x baseline	4-5x baseline	99%
2	7 cases/week 1 hospitalized/week	6/cases week or 3 hospitalized/ week	1.4x baseline 10 cases/week	90%
3	40% of total consultations	3x baseline	4x baseline	95 to 99%
4	Unable to provide a number, but higher than the respiratory syndrome	+20% case increase	N/A	The upper end of the possible range
5	Up to 50% of total consultations	3x baseline	5x baseline	99%
6	15–20% of total consultations	2x baseline	N/A	99%
7	>10% cases/week	Very high increase over the baseline	N/A	Closer to 99%

The table includes participant’s reported baseline of observed cases in their practices.

##### 3.2.7.2 Severity

Participants considered respiratory disease to be less severe than gastrointestinal disease. They also discussed how some dogs’ illnesses are often mislabelled by owners as respiratory disease, e.g., cardiovascular disease. The participants also mentioned how gastroenteric conditions are usually more of a concern for the owners and more intensive and expensive to treat.

“*referring to gastrointestinal disease* this takes more time, it worries me more and it’s more expensive for the owner as well. They’re also more worried, I mean, a sick dog, with diarrhoea and vomiting, for the owner, it’s a very big concern and they come to see us very quickly.”

*— Participant 6: 1.5 years of experience, practice of 2 veterinarians.*

##### 3.2.7.3 Clinically relevant threshold

The excess incidence values were also much higher in the case of syndromes compared to canine pathogens. Most participants provided values for the notification and outbreak response thresholds that ranged between 2 and 12 times over the baseline (see Tables 6, 7). The predictive certainty value was also the highest among canine syndromes and was set to the values of 95 to 99% for both respiratory and gastrointestinal diseases by most participants (see Tables 6, 7). Figure 2 shows an overview of the clinically relevant notification threshold values for canine respiratory and gastrointestinal diseases.

## 4 Discussion

To our knowledge, this is the first study that explores clinically relevant thresholds of case incidence and predictive certainty at which veterinary practitioners would want to be notified about potential outbreaks of canine disease. These clinically relevant thresholds represent veterinarians’ opinions on which outbreak events would be impactful in practice and therefore warrant either being notified about disease anomalies in their area (notification threshold) or triggering an outbreak response (outbreak response threshold). Overall, we found that canine syndromes had higher preferred values of excess case incidence and predictive certainty for the notification and outbreak response thresholds compared to specific canine diseases. Exotic diseases, such as leishmaniosis and babesiosis, had the lowest values of excess case incidence, often totalling to a single case per month to trigger a notification and to change their behavior in practice, as participants perceived that exotic disease outbreaks are likely to be potentially impactful to their practices. Participants’ approaches differed when exploring the predictive certainty of canine endemic diseases, as some of them wanted the highest possible values to avoid false outbreak notifications, while others preferred to keep this parameter at relatively low values to avoid missing out on potential clinically relevant outbreaks or in the case of false alerts to be reminded of the risks that canine infectious diseases can pose to their practices. The findings from the interviews with veterinary practitioners also allowed us to gain an understanding of how the behavior of veterinary clinicians is impacted by outbreaks of canine disease, as included in the codes within the “behavior change” theme (Supplementary material S3). For instance, during an outbreak, participants would increase testing practices for infectious diseases, start vaccination campaigns to protect the local dog population, and increase the frequency of communications with their clients to provide advice on preventative measures.

To achieve the aims of this study, we needed to explore the individual perspectives and experiences of small animal veterinary clinicians. Therefore, a qualitative methodology, consisting of structured and semi-structured interviews, was followed (24). Interview transcripts were analyzed using a hybrid approach to thematic analysis (25). This qualitative methodology is a novel approach to exploring veterinarians’ experiences with canine disease outbreaks, although it has been previously employed in the fields of livestock health (26) and human health (27). The methodology developed in this study was applied to six canine diseases and syndromes that had been identified in a previous study (11) as the top surveillance priorities in the United Kingdom. All participants satisfactorily completed the interviews, and positive feedback was received regarding the usefulness and levels of engagement of the exercise. The information gathered from participants through both types of interviews was rich and allowed us to successfully complete the aims of the study. Thus, this study demonstrates a workable methodology to gain an understanding of which canine outbreak scenarios are relevant to veterinary practitioners and to define their corresponding clinically relevant outbreak notification thresholds.

For infectious diseases, most participants elicited low levels of predictive certainty at given notification thresholds to prioritize sensitivity over the specificity of an outbreak detection system. This risk-averse attitude will ultimately increase the number of outbreak alerts and the proportion of false alerts generated by the system. Most participants argued that they would rather receive false alerts for potential outbreaks that they consider clinically relevant than missing out on relevant information. Some participants even argued that eventually receiving false alerts would be useful for them to be reminded of potential epidemic threats, to improve their epidemic preparedness, and to include infectious causes in their differential diagnosis list. These findings were based on participants’ responses to hypothetical disease outbreak scenarios rather than on practical experience from dealing with actual outbreaks in settings where an alert system had previously been established. We are aware that the outbreak detection systems that generate a high proportion of false alerts could lead end users to a loss of confidence and trust in the system (28). Only by testing this study’s clinically relevant threshold for notification of outbreaks in real-world applications will we be able to understand whether they strike the right balance between sensitivity and specificity.

Overall, notification thresholds for specific infectious pathogens were set at very low levels of excess case incidence, which means that they would like to be alerted of disease anomalies at very low levels of risk. Thus, participants perceived the diseases in this study could represent an epidemic threat to their practices, which is not surprising since such diseases correspond to the top-priority canine diseases for surveillance in the United Kingdom, according to their impact on canine and public health, as found in a previously conducted study (11). The outbreak response threshold values were generally set to greater increases in case incidence than those of the notification thresholds. However, for certain diseases, the notification threshold values provided by some participants often overlapped with the values chosen for outbreak response thresholds by other participants. The reasons for this overlapping may relate to the variation in participant’s perceptions of risk and characteristics of their practice. The variation in participant responses resulted in different ranges of values for both the notification and the outbreak response thresholds, which were wider for some diseases than for others, e.g., the outbreak response threshold for gastroenteric disease ranged from 4 to 5 times over the baseline, while, for respiratory disease, this threshold ranged from 2 to 12 times over the baseline. Although the specific reasons for this variation are unknown, it might be due to the fact that the impacts in veterinary practice of certain diseases are similarly perceived by veterinarians, while other diseases’ impacts are not easily measurable by participants, therefore resulting in a wider range of opinions.

When discussing exotic canine diseases, both the notification thresholds and the predictive certainty values were almost always set to the lowest possible values. Participants also opted to not provide an outbreak notification threshold for the exotic diseases included in this study as they considered that the excess incidence levels of the notification threshold would be enough for them to take action and change their behavior in practice to respond to a potential outbreak. All of these factors indicated that participants perceived exotic disease outbreaks as potentially highly impactful to their practices. This might be because, as observed during the interviews, exotic diseases were perceived as very severe threats despite their epidemiological characteristics and treatment being not well known among veterinary clinicians. According to the decision theory, when making decisions that involve both high risk and high uncertainty, people were more likely to take on a conservative approach and overestimate the risk rather than underestimate it (29). However, as these diseases were not perceived as an immediate threat, participants also reported hardly ever thinking about them or carrying out any preventative actions. Similar attitudes were observed in a previous study where first-line practitioners were interviewed about their experiences with exotic equine diseases (30). In this study, participants reportedly presented a “firefighting approach” to veterinary medicine, where most of the time and effort were spent on immediate threats rather than on preventive or preparedness activities. While not providing an outbreak response threshold for exotic diseases, some participants did make the distinction between autochthonous and imported cases. The threshold value for imported cases was set at higher levels, as participants considered these cases to be sporadic, unrelated events that would not result in an outbreak, as the vectors of the disease are not currently present in the United Kingdom.

We propose an innovative methodology that uses veterinary practitioners’ opinions to inform the selection of a notification threshold value in genuine applications of the stochastic canine outbreak detection models. An advantage of our approach is that it allows us to choose notification thresholds tailored to meet the needs of end users of a surveillance system (i.e., veterinary surgeons in practice). Reducing the proportion of outbreak alerts that are not actionable in clinical settings helps to prevent overloading veterinarians with unnecessary surveillance information while keeping their confidence in such a system. In contrast, the outbreak notification thresholds determined by existing statistical methods (31, 32) often alert end users about genuine statistical signals that are of no practical importance to health professionals. Another strength of the methodology developed in this study is that it can be applied to any pathogen or disease of interest so that it can be adapted to the epidemiological characteristics of any given region.

The clinically relevant thresholds derived from participants’ needs together with the contextual information gained from the qualitative interviews about participants’ experiences with disease outbreaks are intended to be used by SAVSNET as a guide to determine when to notify United Kingdom veterinary practitioners of potential outbreaks. The notification step will be a crucial step for the addition of veterinary clinician input into canine outbreak detection and notification, thus bridging the gap between end users and statistical data.

This study was limited by the number of participating veterinarians, due to the difficulties faced in the recruitment process. The conduction of this study coincided with the peak of the COVID-19 pandemic, which had an overwhelming impact on small animal veterinary practices (33). Furthermore, the number of pet-owning homes in the United Kingdom has significantly increased over the last few years (34), while the number of registered veterinarians in the United Kingdom has not increased at the same rate, partly because of Brexit (35). All these factors have contributed to an increase in the workload of veterinary clinicians, which hindered the recruitment for the study. Indeed, many of the veterinarians who were contacted during the recruitment process reported being interested in the project but having no time to spare to participate. Despite the limited number of participants, their varied backgrounds offered a rich insight into the opinions of veterinary professionals in the United Kingdom. Furthermore, personal experiences are subjective, and it is possible that participants incurred memory bias when recounting past events. The authors strived to compensate for these issues by immersing the participants in outbreak scenarios and asking them repeatedly to reflect and consider the impacts that such outbreaks could have in their practice, given the increased workload, zoonotic risk, and client communications.

In conclusion, this study constitutes a proof of concept and describes a qualitative methodology to define clinically relevant notification thresholds for canine disease outbreaks that are informed by veterinary clinicians and correspond to outbreaks with a significant impact on clinical practice. The methodology has been applied to six top-priority canine diseases and syndromes. Clinically relevant thresholds included a notification threshold and an outbreak response threshold, which represented increases in case incidence that would warrant an outbreak alert or activate an outbreak response, respectively. To the authors’ knowledge, this is the first study that consults end users of a disease surveillance and outbreak notification system (i.e., veterinary clinicians) about their preferences for notification’s excess case incidence and predictive certainty levels. The findings from this study indicate that the developed methodology is adequate to elicit the end-user opinion to establish clinically relevant outbreak alert thresholds. Future studies that apply this methodology should include a larger sample of participants to deepen the understanding of how veterinary clinicians’ preferences vary depending on their experience and background so that outbreak alert thresholds are representatives of the population of companion animal veterinarians in the United Kingdom. The clinically relevant thresholds derived from the needs of veterinary practitioners participating in this study will be used by SAVSNET to inform its outbreak detection system and increase its utility as a strategic informant on the clinical relevance of disease outbreaks in the canine population across the United Kingdom.

## Data availability statement

The original contributions presented in the study are included in the article/Supplementary material, further inquiries can be directed to the corresponding author.

## Author contributions

CT: Conceptualization, Data curation, Formal analysis, Investigation, Methodology, Resources, Software, Validation, Writing – original draft, Writing – review & editing. ES: Supervision, Writing – review & editing. AR: Funding acquisition, Project administration, Resources, Writing – review & editing. JN: Writing – review & editing. FS-V: Conceptualization, Funding acquisition, Methodology, Resources, Supervision, Validation, Writing – original draft, Writing – review & editing.
