# Supplementary material for: Setting clinically relevant thresholds for the notification of canine disease outbreaks to veterinary practitioners: an exploratory qualitative interview study
Source: Front Vet Sci. 2024 Feb 28;11:1259021. doi: 10.3389/fvets.2024.1259021 (PMC10936540; doi:10.3389/fvets.2024.1259021)
Supplement: Supplementary file 1 [file Table_1.DOCX]

## Supplementary Material 1: Topic guide for the semi-structured interview

- What is your experience with disease X? Do you see cases of it in your practice? If so, how many cases would you expect to see in a normal week/month/year? What is the severity of the cases like that you have observed in your practice?
- Are you aware of any exotic canine diseases that could potentially affect you practice? Do you believe there to be enough training or knowledge available to deal with exotic disease cases? What do you think about the risk of introduction of exotic canine pathogens into the UK?
- Have you ever experienced an outbreak of disease X? if so:
  - What did you/ your practice as an institution do to deal with this outbreak?
  - What was your strategy of communication with other veterinarians, the practice’s clients and/or the public?
  - Did you receive any help from other institutions, such as government agencies, veterinary corporations, laboratories, or other?
  - Were there any protocols in place to deal with canine epidemics in your practice? If so, did you follow them at the time, and did you find that they were efficient for dealing with the outbreak?
  - Are there any lessons that you learned from that experience that you would implement in the future if you were faced with a similar situation?

## Supplementary Material 2: Topic guide for the structured interview

- Given what we have discussed so far, could you tell me how many cases of disease X you would need to see in your practice for you to become concerned? Please use a time frame that you are comfortable with, for example in a week, or a month.
- What number of cases in your area would be worrying for you so that you would want to be notified about a potential outbreak?
- How would your behaviour change in response to an outbreak alert of disease X?
- How do you think that a false alarm related to disease X would impact your practice?
- Could you think of an alert certainty level that you would be comfortable with for outbreaks of disease X?

## Supplementary Material 3: Codebook with the codes developed in this study to analyse interview transcripts, both deductively and inductively.

| Babesia | The interviewee talks about any topic that is related to canine babesiosis | 7 | 7 |
| --- | --- | --- | --- |
| Behaviour change | Interviewee describes the actions they would take to respond to an outbreak of canine disease if it were to occur in their current veterinary practice | 3 | 3 |
| Change advice about importation of dogs into the UK | Would change the legislation about the importation of pet dogs into the UK to include more strict measures of testing and quarantining to avoid the introduction of exotic diseases into the country | 3 | 4 |
| Communication with the public | Would attempt to launch a communication strategy with the wider public to share information about the prevention of canine babesiosis | 3 | 4 |
| Contact authorities | Would contact the authorities to notify them of autochthonous/imported cases of canine babesiosis | 2 | 2 |
| Contact diagnostic laboratories and specialists | Would contact diagnostic laboratories and/or veterinary specialists to aid in the diagnosis and/or treatment of canine babesiosis | 2 | 3 |
| Increase testing | Would increase testing in their practice to detect further cases of canine babesiosis | 2 | 4 |
| Information seeking | Would research the clinical presentation/diagnosis/treatment of canine babesiosis to better prepare for potential cases in their practice | 3 | 4 |
| Proactive recommending tick prevention and treatment | Would recommend owners to actively search for ticks in their dogs, as well as preventative actions, such as topical tick prevention, avoiding walks in grassland areas, and using tick repelling collars | 3 | 5 |
| Would not consider contacting authorities | Interviewee explicitly states that they would not try to alert the corresponding authorities of a potential outbreak of canine babesiosis | 1 | 1 |
| Certainty | Interviewee discusses the levels of confidence of an outbreak alert that they would require for a specific canine disease | 7 | 10 |
| Has enough info about the disease | Interviewee believes that the knowledge available in their practice is enough to handle potential cases of canine babesiosis | 1 | 1 |
| Impacts of a false alarm | Interviewee discusses the potential impacts that a false alarm of the disease under discussion would have on their practice | 5 | 5 |
| Costs | Economic impact of the outbreak in their practice | 2 | 2 |
| Information fatigue | Receiving an unnecessarily high number of outbreak alerts, which would result in them becoming desensitised with potential future outbreaks | 2 | 2 |
| No impact | They would not experience any impacts at all from a false outbreak alert | 1 | 1 |
| Positive impact | They would experience a positive effect from false outbreak alerts | 1 | 2 |
| Treatment side effects | They would provide dogs with unnecessary prophylactic treatment, which could result in harmful side effects for the animal | 1 | 1 |
| Imported dogs only | Interviewee discusses only having seen cases of canine babesiosis when imported from other countries into the UK | 4 | 5 |
| Increasing endemic risk | Interviewee perceives the risk of canine babesiosis as increasing as an endemic threat in the UK, for whichever factor(s) | 2 | 2 |
| Lyme disease | Interviewee talks about Lyme disease, in the context of it being a zoonotic disease caused by *Babesia spp,* whether mentioning or not that this is not the same species that causes disease in dogs | 1 | 1 |
| Most worrying disease | Interviewee talks about how canine babesiosis is the disease that worries them the most out of all of the diseases included in Chapter Four | 2 | 4 |
| No clue about babesia | Interviewee mentions how they have never heard about babesiosis as a disease that affects dogs | 1 | 3 |
| Prevalence | Interviewee talks about the prevalence of canine babesiosis in the UK | 7 | 11 |
| Never had a case | Has never seen a case of canine babesiosis in their veterinary practice | 4 | 8 |
| Not more common than Leishmania | Does not consider canine babesiosis as more prevalent than canine leishmaniasis | 3 | 3 |
| Relative risk | Interviewee discusses the levels of disease/increases in case incidence that would result in a behaviour change or that would warrant a notification | 7 | 7 |
| Imported vs non travelled | Makes the distinction between relative risk values chosen for autochthonous and imported cases of canine babesiosis | 3 | 3 |
| Severity of disease | Interviewee discusses the severity of the cases of canine babesiosis | 2 | 2 |
| Suspicion of disease | Interviewee discusses the clinical signs and other risk factors that would make them suspect a potential case of canine babesiosis in veterinary practice | 3 | 5 |
| Ticks | Interviewee talks about ticks as the vector for canine babesiosis | 5 | 6 |
| Veterinarian’s knowledge about Babesia | Interviewee provides their opinion about the knowledge of the veterinary profession in the UK about canine babesiosis | 3 | 4 |
| Wonders about zoonotic potential | Interviewee is not sure about whether canine babesiosis is a zoonotic disease | 2 | 2 |
| **Gastroenteric** | The interviewee talks about any topic that is related to canine gastroenteric disease | 7 | 7 |
| Behaviour change | Interviewee describes the actions they would take to respond to an outbreak of canine disease if it were to occur in their current veterinary practice | 7 | 7 |
| Increased testing | Would consider increasing the frequency of testing for gastroenteric infectious agents | 3 | 3 |
| Causative agents | Interviewee talks about potential causative agents of an outbreak of gastrointestinal disease | 4 | 5 |
| Certainty | Interviewee discusses the levels of confidence of an outbreak alert that they would require for a specific canine disease | 7 | 7 |
| High cost to manage (time, money, effort) | Interviewee talks about the impacts that an outbreak of gastroenteric disease would have in their practice | 2 | 3 |
| Impacts of a false alarm | Interviewee discusses the potential impacts that a false alarm of the disease under discussion would have on their practice | 7 | 7 |
| Prevalence | Interviewee talks about the prevalence of canine gastroenteric disease in the UK | 7 | 9 |
| More common than respiratory | Considers that the prevalence of gastrointestinal disease is higher than that of respiratory disease among UK dogs | 7 | 8 |
| Rarely infectious | Has the opinion that the cases of gastrointestinal disease cases observed in their practice are rarely caused by infectious pathogens | 2 | 3 |
| Relative risk | Interviewee discusses the levels of disease/increases in case incidence that would result in a behaviour change or that would warrant a notification | 7 | 8 |
| Severity of cases | Interviewee discusses the severity of the cases of canine gastroenteric disease that they have seen in their practice | 2 | 2 |
| **Leishmania** | The interviewee talks about any topic that is related to canine leishmaniasis | 7 | 7 |
| Behaviour change | Interviewee describes the actions they would take to respond to an outbreak of canine disease if it were to occur in their current veterinary practice | 7 | 7 |
| Client communication | Would modify their frequency/channel of communication with dog owners | 4 | 5 |
| Information seeking | Would seek information about the potential outbreak and/or study the clinical characteristics of the disease to recognise potential cases in their practice | 3 | 3 |
| Investigation of cases | Would conduct an investigation of the potential cases seen in their practice, e.g., by testing, contact tracing, etc. | 3 | 3 |
| Certainty | Interviewee discusses the levels of confidence of an outbreak alert that they would require for a specific canine disease | 7 | 8 |
| Clinical signs | Interviewee discusses clinical signs that they have either observed or that they believe are indicative of the disease | 5 | 6 |
| Difficulties | Interviewee talks about the difficulties that they believe surround the diagnosis, treatment, or prevention of the disease | 3 | 4 |
| Accurately knowing dogs’ travel and vaccination history | Not knowing the history of the animal is considered as a difficulty for the detection of leishmaniosis cases | 1 | 1 |
| Impacts of a false alarm | Interviewee discusses the potential impacts that a false alarm of the disease under discussion would have on their practice | 2 | 3 |
| Costs | Economic impact of epidemic preparedness in their practice | 1 | 1 |
| No impact | False outbreak alerts would not have an impact in their practice | 1 | 2 |
| Not bothered by frequent alerts | False outbreak alerts would not be an inconvenience | 2 | 2 |
| Increasing awareness | Interviewee believes there is an increasing awareness of canine leishmaniosis by the veterinary profession | 1 | 1 |
| Low prevalence | Interviewee believes that the prevalence of canine leishmaniosis is very low in the UK | 4 | 6 |
| Never diagnosed a case | Has never diagnosed a case | 2 | 2 |
| Only seen imported cases | Has only seen cases that were imported from other countries | 5 | 7 |
| Aware of non-travelled cases | Is aware that autochthonous cases of canine leishmaniosis have been reported in the UK | 1 | 1 |
| Misconceptions | Participant’s misconceptions regarding the transmission and characteristics of canine leishmaniosis | 3 | 5 |
| Preventative action | Interviewee describes existing protocols used in their veterinary practice to prevent outbreaks of canine disease | 1 | 1 |
| Relative risk | Interviewee discusses the levels of disease/increases in case incidence that would result in a behaviour change or that would warrant a notification | 7 | 10 |
| Imported vs non travelled dogs | Interviewee makes a distinction in the relative risk levels chosen for the notification and high alert thresholds for canine leishmaniosis | 3 | 3 |
| Sandfly | Interviewee mentions the vector for canine leishmaniosis | 4 | 6 |
| Screening and import refusal | Interviewee discusses screening protocols for canine leishmaniosis | 1 | 1 |
| Severity of disease | Interviewee talks about the severity of canine leishmaniosis | 1 | 1 |
| Uncertainty about treatment, diagnosis, monitoring | Interviewee discusses the uncertainties surrounding the diagnosis, treatment, or prevention of the disease | 4 | 7 |
| Unlikely to be transmissible | Interviewee does not think that leishmaniosis is a highly transmissible disease | 3 | 4 |
| Not concerned about epidemic potential | Is not concerned about leishmaniosis as an outbreak-causing pathogen | 1 | 5 |
| Vaccination | Interviewee talks about the vaccination of canine leishmaniosis | 3 | 4 |
| Very concerned about it becoming endemic | Interviewee expresses concern about the potential endemisation of canine leishmaniosis in the UK | 2 | 3 |
| Climate change | Impact of climate change in the likelihood of endemisation of canine leishmaniosis in the UK | 2 | 2 |
| Zoonotic potential | Interviewee talks about the zoonotic potential of canine leishmaniosis | 5 | 7 |
| **Leptospirosis** | The interviewee talks about any topic that is related to canine leptospirosis | 7 | 8 |
| Behaviour change | Interviewee describes the actions they would take to respond to an outbreak of canine disease if it were to occur in their current veterinary practice | 4 | 4 |
| Active investigation | Would investigate of the leptospirosis cases seen in practice, e.g., through contact tracing | 3 | 3 |
| Costs covered by practice | The costs of responding to the outbreak would be covered by their practice | 1 | 1 |
| Biosecurity measures | What biosecurity measures they would put in place to prevent the spread of the outbreak | 3 | 7 |
| Communication strategy | Talks about whether or not they would start a communication campaign during an outbreak of canine leptospirosis | 2 | 3 |
| Advice to clients | Would provide advice to clients on how to prevent the disease | 3 | 6 |
| Didn't contact anyone | Did not contact anyone during a past outbreak of canine leptospirosis | 1 | 1 |
| Don't know who I would contact | Does not know who they would contact during an outbreak of canine leptospirosis | 3 | 4 |
| Social media | Would use social media to post about the outbreak | 2 | 2 |
| Vaccination companies | Would get in touch with vaccination companies | 1 | 1 |
| With other vets | Would attempt to contact other veterinarians in the area | 2 | 2 |
| Increase testing | Would increase testing to detect further cases of leptospirosis | 3 | 4 |
| Information seeking | Would seek information about the status of the outbreak or about the characteristics of the disease | 3 | 3 |
| Knowledge within own practice | Would check the available knowledge within their own practice | 1 | 2 |
| MSD | Would use the resources provided by MSD | 1 | 1 |
| Vet voices | Would seek information using the Facebook group “vet voices” | 1 | 1 |
| Isolation and movement control | Would recommend isolation of and movement restrictions of cases | 1 | 1 |
| Not much can be done | Interviewee believes that not many actions could be taken to respond to an outbreak of canine leptospirosis | 1 | 1 |
| Recommend vaccination | Would recommend dog owners to vaccinate their animals | 3 | 4 |
| Unlikely to do financial preparedness |  | 1 | 1 |
| Unsure what to do | Would not know what to do | 1 | 1 |
| Certainty | Interviewee discusses the levels of confidence of an outbreak alert that they would require for a specific canine disease | 7 | 16 |
| Diagnosis | Interviewee talks about the diagnosis of canine leptospirosis | 3 | 3 |
| Difficulties | Difficulties surrounding the diagnosis of the disease | 4 | 7 |
| Necropsy | Necropsies to confirm the diagnosis of leptospirosis | 1 | 2 |
| Not confirmed | Suspected cases of canine leptospirosis were not diagnostically confirmed | 3 | 8 |
| Serology vs PCR testing | Compares different diagnostic methods for canine leptospirosis | 2 | 3 |
| Experience with outbreak | Interviewees who were involved in outbreaks of canine leptospirosis discuss their experiences | 3 | 3 |
| Case load | Case incidence of canine leptospirosis during the outbreak | 3 | 4 |
| Clinical presentation | Clinical presentation the observed cases during the outbreak | 2 | 5 |
| Concerns about zoonotic potential | Concerns about the zoonotic potential of leptospirosis during the outbreak | 2 | 3 |
| Lessons learned | Lessons learned during the outbreak of canine leptospirosis that participants would apply in future outbreaks | 2 | 16 |
| Duration | Duration of the outbreak of leptospirosis | 2 | 2 |
| Emotions | Emotions expressed by the participant in relation to how they felt during the outbreak of canine leptospirosis | 2 | 6 |
| Impacts of an outbreak | Interviewee discusses the impacts that the outbreak of leptospirosis had in their practice | 1 | 2 |
| Economic impact | Financial impacts of the outbreak | 2 | 8 |
| Higher workload | Increased workload for the veterinarians employed at their practice | 2 | 4 |
| Mental health | Impact on staff’s wellbeing and mental health | 2 | 3 |
| Training | Need to invest in staff training to handle the leptospirosis cases during the outbreak | 1 | 1 |
| Information sources | Information sources that participants used during the outbreak of leptospirosis | 2 | 2 |
| Response | Actions that were taken by participants during the outbreak of leptospirosis to handle the outbreak | 1 | 2 |
| Biosecurity | Increased biosecurity measures | 2 | 5 |
| Communications | Communications that participants carried out during the outbreak of leptospirosis | 2 | 12 |
| Media | Interactions with the media | 2 | 3 |
| Owners | Interactions with dog owners | 1 | 1 |
| Participating institutions | Contact with institutions that provided advice/resources during the outbreak | 2 | 5 |
| Public perception | Opinions that members of the public shared with participants during the outbreak | 2 | 4 |
| Success of communications | Whether communications were successful during the outbreak | 2 | 2 |
| With referring vets | Communications with veterinarians that referred the cases of leptospirosis to participant’s practice during the outbreak | 1 | 1 |
| Within practice | Internal communications during the leptospirosis outbreak | 1 | 1 |
| Proactive treatment | Proactively treating highly suspicious cases of leptospirosis | 2 | 3 |
| Testing | Actively testing for the disease when recognising clinical signs | 2 | 7 |
| Vaccination | Started a vaccination campaign during the outbreak to prevent further cases | 2 | 2 |
| Well-coordinated | Interviewee believes that the outbreak response was well-coordinated at the time | 2 | 2 |
| Lack of help | Interviewee talks about how they did not receive any help during the outbreak from external institutions | 2 | 8 |
| Lessons learned | Interviewee discusses the lessons that they learned during their experience with an outbreak with canine leptospirosis | 2 | 3 |
| Negative opinion about the authorities | Interviewee expresses a negative perspective about the authorities, regarding the lack of help provided during the outbreak of leptospirosis | 2 | 4 |
| Outbreak detection | Interviewee talks about how the leptospirosis outbreak was detected | 3 | 10 |
| Outcome | Interviewee talks about the outcome of the cases of canine leptospirosis that they experienced during the outbreak | 4 | 9 |
| Severity of cases | Interviewee talks about the severity of the cases of leptospirosis that they saw during the outbreak | 1 | 2 |
| Sources of funding | Interviewee talks about the sources of funding that they received when responding to the leptospirosis outbreak | 1 | 1 |
| I would certainly want it on the notification system | Direct quote from participant | 1 | 1 |
| Impacts of a false alarm | Interviewee discusses the potential impacts that a false alarm of the disease under discussion would have on their practice | 5 | 7 |
| Explaining to owners | Having to discuss the potential outbreak and its implications with dog owners | 3 | 4 |
| Inappropriate use of antibiotics | Using antibiotics prematurely when suspecting that there is an outbreak | 1 | 1 |
| Increased testing | Covering the costs of testing | 1 | 1 |
| Information fatigue | Receiving too many alerts that are not relevant and becoming disengaged with the alert notification system | 1 | 1 |
| No extra cost | Does not believe that there would be any costs associated to a false outbreak alert | 2 | 3 |
| No impact | Does not believe that there would be any impacts associated to a false outbreak alert | 3 | 4 |
| Positive impact | Participant believes that the impacts of a false alert would be positive for their practice | 2 | 2 |
| Resource investment | Investment in resources for epidemic preparedness | 2 | 2 |
| In dairy farms | Interviewee talks about the cases of canine leptospirosis that they have seen in dairy farms | 1 | 2 |
| Prevalence | Interviewee talks about the prevalence of canine leptospirosis in the UK | 7 | 9 |
| Decreased | Has decreased over recent years | 4 | 7 |
| Very rare | Considers canine leptospirosis as a very rare occurrence in the UK | 6 | 11 |
| Mostly in unvaccinated dogs | Most cases occur in unvaccinated animals | 4 | 6 |
| L4 controversy | Interviewee talks about the controversies associated with the leptospirosis L4 vaccine | 4 | 4 |
| Most important disease | Interviewee considers canine leptospirosis as the most important disease for their practice | 1 | 1 |
| Relative risk | Interviewee discusses the levels of disease/increases in case incidence that would result in a behaviour change or that would warrant a notification | 7 | 20 |
| Risk factors | Interviewee talks about the risk factors for canine leptospirosis | 6 | 11 |
| Severity of disease | Interviewee discusses the severity of the cases of canine leptospirosis | 1 | 2 |
| Suspicion of disease | Interviewee talks about the clinical signs that would make them suspect a case of canine leptospirosis | 5 | 13 |
| Uncommon clinical presentation | Has seen cases with an unusual clinical presentation | 1 | 3 |
| Talked about every day | Participant says that they talk about the risk of canine leptospirosis every day in their practice | 1 | 1 |
| Treatment | Participant talks about the treatment for canine leptospirosis | 3 | 4 |
| Underdiagnosed | Participant believes that the disease is underdiagnosed in the country and that the real prevalence is higher than what we currently estimate | 2 | 4 |
| Vaccination | Participant talks about the vaccination of canine leptospirosis | 5 | 8 |
| Core vaccine | Mentions how leptospirosis is included in the core vaccinations list in the UK | 2 | 2 |
| L4 | Participant mentions the L4 vaccine | 3 | 5 |
| Shortage | Participant talks about the current shortage of leptospirosis vaccines | 1 | 1 |
| Zoonotic risk | Interviewee talks about their perception of the zoonotic risk of canine leptospirosis | 5 | 9 |
| Misconceptions | Misconceptions about canine leptospirosis | 2 | 4 |
| **Parvovirus** | The interviewee talks about any topic that is related to canine parvovirus | 7 | 7 |
| Behaviour change | Interviewee describes the actions they would take to respond to an outbreak of canine disease if it were to occur in their current veterinary practice | 1 | 1 |
| Biosecurity measures | Would implement biosecurity measures | 4 | 8 |
| Communications | Would start a communication campaign | 5 | 8 |
| Increase testing | Would increase testing to detect parvovirus cases | 2 | 3 |
| Proactive treatment | Would proactively treat potential cases of disease to mitigate their severity | 2 | 2 |
| Promote vaccination | Would recommend vaccination to dog owners | 2 | 2 |
| Specific parvo protocol | Participant talks about a specific protocol for canine parvovirus in their practice | 1 | 2 |
| Certainty | Interviewee discusses the levels of confidence of an outbreak alert that they would require for a specific canine disease | 7 | 12 |
| Client concerns | Interviewee talks about their practice’s clients concerns about canine parvovirus | 2 | 3 |
| Difficulties and lessons learned | Interviewee talks about the lessons learned from previous cases of canine parvovirus | 3 | 6 |
| Expensive to treat | Considers parvovirus as an expensive disease to treat | 2 | 2 |
| Fatal outcome due to funds running out | Participant discusses how the disease often results in fatalities due to the lack of funds from dog owners | 2 | 2 |
| Impacts of a false alarm | Interviewee discusses the potential impacts that a false alarm of the disease under discussion would have on their practice | 6 | 7 |
| Costs | Financial impacts of a false alarm | 2 | 2 |
| Low impact | Would not have a very big impact in their practice | 3 | 5 |
| Owner's concern | Would create unnecessary concerns for dog owners who are clients of the practice | 1 | 1 |
| Positive impact | Would have a positive impact in their practice | 1 | 1 |
| Waste of resources | Would result in resources being wasted | 1 | 1 |
| Prevalence | Interviewee talks about the prevalence of canine parvovirus in the UK | 7 | 12 |
| Hearing of outbreaks | Participant reports how it is common to sporadically hear about outbreaks of canine parvovirus in the country | 5 | 7 |
| Not seen over a period of time | Has not seen any cases of the disease in recent times | 3 | 6 |
| Upsurge due to new variants | Has observed an increase in the prevalence of disease, presumably due to newly emerging variants | 1 | 1 |
| Portrayal in the media | Interviewee talks about the media’s portrayal of canine parvovirus | 1 | 1 |
| Relative risk | Interviewee discusses the levels of disease/increases in case incidence that would result in a behaviour change or that would warrant a notification | 7 | 19 |
| Relevance despite non zoonotic | Interviewee stresses how canine parvovirus is an important disease despite it not having a known zoonotic potential | 7 | 7 |
| Risk factors | Interviewee talks about the risk factors for canine parvovirus | 4 | 6 |
| Puppies | Higher prevalence in puppies | 5 | 5 |
| Socioeconomic status | Families’ socioeconomic status | 4 | 5 |
| Urban areas | Dogs living in urban areas | 2 | 2 |
| Vaccination history | Not vaccinated, unclear history, wrong vaccination times, etc | 5 | 8 |
| Severity of disease | Interviewee talks about the severity of canine parvovirus | 2 | 4 |
| Suspicion of disease | Interviewee discusses the clinical signs of canine parvovirus that would make them suspect the disease in practice | 3 | 4 |
| Clinical signs observed | Interviewee talks about the clinical signs of parvovirus that they have observed previously in clinical practice | 4 | 4 |
| Testing and vaccination | Participant discusses the testing and vaccination practices for canine parvovirus in their practice | 4 | 7 |
| **Respiratory** | The interviewee talks about any topic that is related to canine respiratory disease | 7 | 7 |
| Behaviour change | Interviewee describes the actions they would take to respond to an outbreak of canine disease if it were to occur in their current veterinary practice | 2 | 2 |
| Increase awareness only | Would only consider speaking with dog owners to increase their awareness about potential signs of disease | 1 | 1 |
| No extra cost | Does not believe that their practice would incur in extra costs during an outbreak of respiratory disease | 1 | 1 |
| Not much can be done | Does not believe that many actions could be taken to respond to an outbreak of canine parvovirus | 1 | 2 |
| Refer cases | Would refer cases to a veterinary hospital | 1 | 1 |
| Wouldn't consider hiring someone extra | Does not believe that extra staff would be hired to manage the extra workload during an outbreak | 1 | 2 |
| Causative agents | Interviewee talks about the infectious pathogens that cause respiratory disease in dogs | 5 | 6 |
| Usually unknown | Participant discusses how the causative agent of respiratory disease is not normally known, as no tests are performed | 2 | 4 |
| Certainty | Interviewee discusses the levels of confidence of an outbreak alert that they would require for a specific canine disease | 6 | 7 |
| Impacts of a false alarm | Interviewee discusses the potential impacts that a false alarm of the disease under discussion would have on their practice | 4 | 5 |
| Costs | Financial impacts of a false alarm | 1 | 1 |
| No impact | Does not think that a false alarm would have any impacts in their practice | 2 | 3 |
| Low severity | Participant believes that the severity of respiratory disease cases is low | 2 | 2 |
| Overwhelming | Participant discusses how an outbreak of respiratory disease would overwhelm their practice | 3 | 3 |
| Prevalence | Interviewee talks about the prevalence of canine respiratory disease in the UK | 7 | 10 |
| Rarely infectious | Interviewee talks about how the causes are rarely infectious | 2 | 2 |
| Low prevalence (compared to GI) | Interviewee believes that the prevalence of respiratory canine disease is low, especially compared to gastrointestinal disease | 2 | 3 |
| Relative risk | Interviewee discusses the levels of disease/increases in case incidence that would result in a behaviour change or that would warrant a notification | 7 | 12 |
